# Climate Change, Air Quality, and Pollen Allergies—State of the Art and Recommendations for Research and Public Health

**DOI:** 10.1111/all.70159

**Published:** 2025-12-20

**Authors:** Jobst Augustin, Stefan Gilge, Heike Appel, Ute Dauert, Christina Endler, Ruth Heesen, Conny Höflich, Wilhelm Kuttler, Katharina Heinke Schlünzen, Wolfgang Straff, Barbora Werchan, Matthias Werchan, Torsten Zuberbier, Claudia Traidl‐Hoffmann

**Affiliations:** ^1^ Institute for Health Services Research in Dermatology and Nursing (IVDP) University Medical Center Hamburg‐Eppendorf (UKE) Hamburg Germany; ^2^ German Meteorological Service, Climate and Environment Division, Research Center Human Biometeorology Freiburg Germany; ^3^ Department of Otorhinolaryngology, Head and Neck Surgery Ulm University Medical Center (UKU) Ulm Germany; ^4^ Section II 4.2, Air Quality Assessment German Environment Agency Dessau‐Roßlau Germany; ^5^ VDI – The Association of German Engineers VDI/DIN‐Commission on Air Pollution Prevention (KRdL) – Standards Committee Düsseldorf Germany; ^6^ Section II 1.5 Environmental Medicine and Health Effects Assessment German Environment Agency Berlin Germany; ^7^ Faculty of Biology, Applied Climatology and Landscape Ecology University of Duisburg‐Essen Essen Germany; ^8^ Meteorological Institute University of Hamburg Hamburg Germany; ^9^ German Pollen Information Service Foundation (PID) Berlin Germany; ^10^ Institute of Allergology Charité – Universitätsmedizin Berlin, Corporate Member of Freie Universität Berlin and Humboldt‐Universität zu Berlin Berlin Germany; ^11^ Fraunhofer Institute for Translational Medicine and Pharmacology ITMP, Immunology and Allergology Charité – Universitätsmedizin Berlin Berlin Germany; ^12^ Institute of Environmental Medicine and Integrative Health, Faculty of Medicine University Augsburg Augsburg Germany; ^13^ Institute of Environmental Medicine, Environmental Health Center Helmholtz Munich Munich Germany

**Keywords:** air pollution, airborne pollen, allergic rhinitis, meteorology, policy, stakeholder

## Abstract

Allergies are one of the major health challenges of our time, associated with a high individual burden of disease and high costs for the healthcare system. Given their prevalence, allergies are also highly relevant from a public health perspective. The development of allergic diseases is multifactorial. In addition to individual factors (e.g., genetic predisposition), environmental factors are particularly important. These include climate (including climate change), weather, and air pollution, which affect the biosphere and biodiversity. Pollen‐associated allergic rhinitis is one of the most common allergies. Airborne pollen is strongly connected with climate (change) and air pollution. For example, interannual climate variability and climate change affect phenology, pollen production, and pollen transport, and air pollutants affect pollen allergenicity. Climate change also affects air quality as meteorological conditions influence relevant processes such as the emission, transport, chemistry, and deposition of air pollutants, which affect the occurrence, intensity, and duration of allergy symptoms. The aims of this position paper are: (a) to provide an overview of the current state of scientific knowledge on the effects of climate change and air quality on pollen allergies, (b) to discuss conflicting objectives in the fight against pollen allergies, and (c) to provide recommendations for policy makers, health professionals, public health measures, and future research.

## 1 Introduction

Allergies are one of the major health challenges of our time, and allergic diseases are associated with a high individual burden of disease as well as high direct and indirect costs for the healthcare systems [1, 2, 3, 4, 5]. Allergic rhinitis belongs to the most frequent allergies. For example, in Germany, about 15% of adults and 11% of children suffer from this disease (lifetime prevalence), corresponding to more than 10 million people, with airborne pollen being one of the main allergens causing allergic rhinitis [6]. Apart from being a direct allergy trigger, pollen also seems to increase susceptibility to viral infections of the respiratory tract [7, 8] and to non‐allergic rhinitis [9].

The occurrence of allergic diseases in general and allergic rhinitis in particular is associated with several factors. On the one hand, individual determinants (e.g., genetic predisposition, lifestyle) could lead to an allergic disease. On the other hand, environmental factors contribute to its development, including climate (incl. climate change), weather, and air pollution, having an impact among others on the biosphere and biodiversity [10].

Climate is defined as “the statistical description in terms of mean and variability of relevant quantities over a period of time. (…) The classical period (…) is 30 years (…). The relevant quantities are most often surface (…)” [11]. Atmospheric parameters related to temperature, pressure, wind, humidity, and precipitation. Climate does not have a spatial definition but covers local, regional, and global scales. Climate change can be defined as “a change in climate which is attributed directly or indirectly to human activity that alters the composition of the global atmosphere and that is in addition to natural climate variability observed over comparable time periods” [12].

The interannual variability of climate and climate change has effects on pollen, such as variations in phenology, in pollen production, and allergenicity, in pollen transport and dispersion, and in the spread of plants (with allergy‐relevant pollen) to new areas [13, 14, 15]. Exposure to pollen from these spreading plants—whether native, non‐native or invasive plants—can lead to sensitisation in populations that have not previously reacted to these plants [15]. In Germany, this may already apply to the pollen of the potentially invasive common ragweed ( *Ambrosia artemisiifolia* ) and in the near future also for instance to pollen from the olive tree ( *Olea europaea* ) [16] or the invasive tree‐of‐heaven ( *Ailanthus altissima* ) [17]. Besides this, rising carbon dioxide (CO_2_) concentrations could increase the allergen concentration of pollen, as shown for Amb a 1 in *Ambrosia artemisiifolia* pollen [13, 18, 19].

Climate change also has an impact on air quality as meteorological conditions influence relevant processes such as emissions, transport, chemistry, and deposition of air pollutants (e.g., NO_x_, O_3_, PM) and their precursors [20]. The resulting change in the composition and/or concentration of air pollutants is likely to have an impact on the occurrence, intensity, and duration of allergy symptoms, for instance, by effects of air pollutants on pollen‐producing plants [21, 22], by interactions between air pollutants and pollen [23, 24, 25], or by effects of air pollutants on skin barriers [26, 27].

Climate change, pollen, and air pollution affect human health in most regions of the globe. In urban areas, however, the effects are exacerbated for a number of reasons: (a) enhanced night‐time temperatures (urban heat island effect) due to densely built urban areas [28], (b) reduced evaporation and ground water availability due to the sealed surfaces as well as reduced average wind speed and enhanced gustiness by the buildings' influences, and (c) reduced air quality in urban areas due to higher emissions resulting from human activities and urban climate [29]. As a result, more attention is being paid to urban areas in many regions, for example, to reduce urban overheating by creating more green spaces, which can have both positive and negative effects on human health.

**FIGURE 1:**
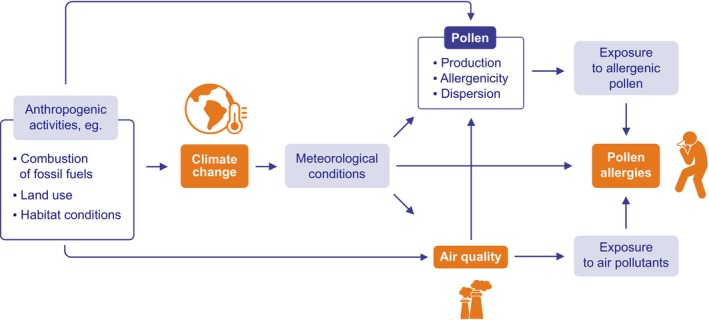
Schematic depiction to describe the connection between climate, pollen, air pollutants and pollen allergies. NO_x_, Nitrogen oxides; O_3_, Ozone; PM, Particulate Matter; VOC, Volatile Organic Compounds.

The association between climate (change), pollen allergies, and air pollutants is shown schematically in Figure 1. Pollen contributes directly, and air pollutants contribute directly or indirectly to the onset and aggravation of pollen allergies [30, 31, 32], and both pollen and air pollutants are affected by changes in climate. Exposure to pollen (and air pollutants) should be mentioned, as it is related to the severity of symptoms [33].

Besides pollen, airborne fungal spores also trigger allergies [34]. Because of the limited number of studies on the impact of climate change and air pollution on fungal spores and allergy [35, 36, 37], this article does not deal with fungal spores. Nevertheless, the authors of this article would like to point out the urgent need for more research in this area.

The aim of this position paper is to provide an overview of the current state of scientific knowledge on the effects of climate change and air quality on pollen allergies. Influences of the meteorological factors affected by climate change on pollen allergies are summarised in Section 2. Influences of climate change on air quality are summarised in Section 3. The effect of air quality on pollen allergic diseases is discussed in Section 4. Section 5 compares specific urban and rural areas regarding pollen allergies. Finally, conflicting goals for combating pollen allergies are discussed in Section 6, and recommendations for political actors and health experts, as well as for future research, are given in Section 7.

## 2 Climate Change Affecting Pollen Allergies

### 2.1 Changes in Phenology and Pollen Season

Phenology is the timing of seasonal events such as bud burst, flowering, dormancy, migration, and also hibernation [38]. The timing of flowering and pollination depends on the current meteorological conditions and those of previous months. The most important factors influencing plant development in the mid‐latitudes are air temperature (especially temperature sum) and day length. A warmer climate may shift the flowering period to an earlier start [24] and extend the pollen season of some plant species [14, 39, 40]. On the other hand, temperate plants often require a certain exposure to chilling temperatures during winter to release endogenous dormancy [41]. Afterwards, the vegetative bud bursts, or flowering will be triggered by a sufficient exposure to forcing temperature [42]. As warming affects both the chilling and forcing exposure, their interactive effects on the timing of flowering may cause ambiguous responses [43, 44]. Many previous studies attribute advances in bud burst to increased forcing [45, 46, 47]. Ettinger et al. [43] found that delays in phenology due to decreased chilling only occur at warming above at least 4°C. However, in the Mediterranean region (Spain), there is already a tendency for the alder pollen season to start later, which coincides with autumn warming [48].

Table S1 summarises results for the onset of flowering of selected plant taxa or the beginning of the pollen season of selected pollen types in different regions. Table S2 gives examples of changes in the Annual or Seasonal Pollen Integral (APIn or SPIn). The Annual or Seasonal Pollen Integral indicates the total amount of pollen in the air over a given period of time, typically a year or a season [49]. The results of pollen season trend analyses are influenced by the definition of pollen season used [40, 50, 51]. When comparing phenological data and pollen concentration in the air, it is important to keep in mind that the occurrence of pollen grains in a particular region is not necessarily related to the local flowering period. In Central Europe, for example, the appearance of birch pollen in the air and thus the start of the birch pollen season may precede the onset of flowering of local birch stands by several days or even weeks. The reason for this is usually the pre‐seasonal long‐distance transport of pollen from regions with an earlier start of birch flowering [52]. Consequently, for a given region, changes in phenological data cannot be transferred one‐to‐one to variations in the pollen season. Climate change is clearly contributing to a shift in the flowering phenology of trees and herbaceous plants. Pollen from trees tends to appear earlier and pollen from herbaceous plants later in the year than in the past, resulting in a potentially longer pollen exposure [53]. Against the backdrop of changes in phenology and pollen dispersal, it is particularly important to develop innovative methods (e.g., Makra et al. [54]) for predicting pollen exposure in order to prevent pollen allergy sufferers from being exposed to high concentrations of airborne allergens in the future [55].

### 2.2 Effects on Pollen Production

Pollen production is plant species‐specific and is influenced by multiple factors, for example, the type of pollination [56] plant size at the beginning of flowering [57] or environmental or stress factors throughout the life cycle of the plant [58, 59]. Therefore, differences in pollen production can also occur within the same species. For example, individuals of some species show natural fluctuations in pollen production over time—e.g., beech (*Fagus*) [60] or ash (*Fraxinus*) [61]. Regarding weather and climate conditions, low temperature and drought limit the plant size and pollen production of ragweed ( *Ambrosia artemisiifolia* ) [62]; for groundnut ( *Arachis hypogaea* ), temperatures higher than 34°C during a 6‐day stress period had a negative effect on pollen production [63]. On the other hand, an increase in carbon dioxide and nitrogen dioxide concentrations in the air leads to increased ragweed pollen production [64, 65, 66]. Especially higher temperatures and CO_2_ concentrations were linked to higher levels of pollen production in various plant species [67, 68, 69]. Male plants of juniper ( *Juniperus communis* ) and yew ( *Taxus baccata* ) grown in a nutrient‐rich environment produced more pollen, but of reduced quality (lower in vitro germination potential and smaller pollen grain volume) than plants grown without any fertilisation [59, 70]. Ranpal et al. [71] reported considerable differences in pollen production among same‐aged birch trees growing under similar microclimatic conditions in a small geographic area. In another recent study, downy birch pollen production across Europe was assessed using genetically identical plants collected from 2019 to 2021 [72]. The study evaluated the impact of meteorology (temperature and precipitation) and atmospheric gases (ozone (O_3_) and carbon dioxide (CO_2_)) on pollen and catkin production. The results showed significant geographic variability in pollen production, ranging from 1.9 to 2.5 million grains per catkin. Higher average temperatures from the previous summer increased pollen production, whereas higher O_3_ levels reduced it. Catkin numbers were positively influenced by the preceding summer's temperature and precipitation, but negatively affected by O_3_ [72]. This study highlights the potential impacts of climate change on downy birch pollen production, crucial for birch reproduction and human health. In addition, it has to be considered that plant viral infections also have an impact on the quantity of birch pollen production; namely, a significantly lower amount of pollen was found in catkins with virus‐infected pollen [73].

### 2.3 Effects on Pollen Dispersion

Pollen dispersal ability is primarily determined by a plant's mode of pollination, with both the plant and its pollen adapted to a specific pollination strategy. From an allergological perspective, zoophilous and anemophilous plants are relevant, although the latter are of significantly greater importance in this context. Zoophily, i.e., pollen transfer by animals (e.g., entomophily—insects; ornithophily—birds), is the most common type of pollination. Pollen from zoophilous plants is usually rarely found in the air. Nevertheless, there are certain zoophilous plants whose pollen can also be a common component of the airborne pollen spectrum up to pollen taxon‐specific concentrations sufficient to cause sensitisation or allergic symptoms in the exposed population—for example, willow (*Salix*) [74], lime tree (*Tilia*) [75], or tree‐of‐heaven (*Ailanthus*) [76]. In the case of *Ailanthus altissima*, it is discussed that this species is not exclusively entomophilous, but ambophilous (wind and insect pollinated). The most allergy‐relevant pollen is found among anemophilous plants whose pollen is transferred by wind—for example, grass family (*Poaceae*), birch (*Betula*), alder (*Alnus*), ragweed (*Ambrosia*), pellitory (*Parietaria*), or cypress family (*Cupressaceae*). Many anemophilous tree species [e.g., sessile oak ( *Quercus petraea* )] and shrubs [e.g., common hazel ( *Corylus avellana* )] flower before the foliage, which would otherwise reduce pollen dispersal. Therefore, late winter and early spring are periods with a prevalence of pollen allergies caused by tree and shrub pollen.

The distances between pollen source and receptor point where the pollen reaches the ground cover a wide range from a few meters to 1000 km and more [77, 78, 79, 80]. The range of pollen dispersal distance is influenced by the type of pollination, the time of flowering (flowering before or during full foliage), the meteorological conditions at the time of emission and along the trajectory of dispersion, the spatial distribution of the sources, the number, size, shape, and weight (deposition rate) of the emitted pollen, and the proximity of dispersal obstacles such as neighbouring vegetation or buildings. In addition, various meteorological parameters are relevant, in particular wind, relative humidity, precipitation, and atmospheric stability (convective or stable atmosphere). Pollen may also influence meteorological conditions: Pollen and subpollen particles enhance cloud formation and can act as ice nuclei in the atmosphere (e.g., Gute and Abbatt) [81], affecting precipitation and pollen transport distance.

Climate change increases the energy in the atmosphere [82], which is likely to intensify atmospheric conditions that favour the dispersion and transport of larger airborne particles (e.g., heat‐induced updrafts) [83] and droughts [84]. Climate projection models should be used to investigate whether this will lead to a wider, more uniform distribution (assuming constant emissions) with correspondingly lower regional concentrations, or to an accumulation in certain favoured regions.

### 2.4 Changes in the Regional Spectrum of Plants With (Potentially) Allergy‐Relevant Pollen

**FIGURE 2:**
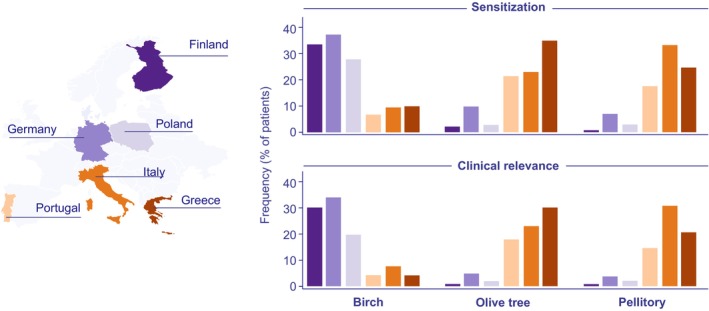
Sensitisation and respective clinical relevance rates of pollen from birch, olive tree, and pellitory in selected European countries, on the basis of data from Burbach et al. [88] (study population: Outpatients with suspected present or former allergic reaction to inhalant allergens, total of valid data sets analysed: *N* = 3034, study period: 2007/2008).

The impact of changes in the regional plant spectrum due to global warming varies by plant species and geographical location. So, global warming may affect the distribution of various plant species by shifting their ranges to higher elevations or higher latitudes [85] and therefore may cause striking spatial and temporal variability in the pollen season [86]. As a result, some plants with allergy‐relevant pollen may lose their current habitats and through this their regional clinical relevance in the coming decades (such as birch in Bavaria, Germany) [87], whereas plants typical of the Mediterranean region, such as olive tree (*Olea*) or pellitory (*Parietaria*), may spread to higher latitudes and cause an increase in sensitisation rates in these regions. Currently, in European patients with respiratory allergies sensitisation and clinical relevance rates of olive tree ( *Olea europaea* ) or pellitory (*Parietaria*) are relatively high in Mediterranean countries such as Portugal, Italy, or Greece, but rather low in more northern countries such as Finland, Germany, or Poland, and the opposite is observed for birch (*Betula*) (Figure 2) [88].

In summary, because of climate change, cold‐adapted plants may lose their current habitats, whereas warm‐adapted plants are likely to continue to expand. Besides the olive tree ( *Olea europaea* ) and pellitory (*Parietaria*), ragweed (*Ambrosia* spp.) or tree‐of‐heaven ( *Ailanthus altissima* ) are other plant species to which this may apply.

#### 2.4.1 Ragweed: A Potentially Invasive Plant With High Allergenic Potential

Ragweed (*Ambrosia* spp.), originally native to North America, has been imported as an agricultural product or spread through contaminated birdseed and is already established in some regions of Europe, such as Hungary, Italy (Po Valley), France (Rhone Valley), and some regions of Germany [89, 90, 91].

Because of climate change, a significant increase in its habitat suitability has been projected for the coming decades, and consequently, the number of sensitised people in Europe would increase [15, 91, 92]. The largest proportional increase would occur in regions where sensitisation is uncommon today.

Ziska et al. [93] showed that ragweed in an urban area with high CO_2_ concentrations grew faster and flowered earlier and more intensely, resulting in higher pollen production, as compared to ragweed grown in an adjacent rural area. Furthermore, the allergenicity of ragweed pollen can be increased by higher atmospheric CO_2_ levels and increased drought [94].

### 2.5 Effects on Thunderstorm Asthma

The local meteorological situation has an important influence on the regional occurrence of pollen (Section 2.3) and thus on allergy symptoms. One example is the so‐called Thunderstorm Asthma (TA) [95]. TA is a rare but serious event with an emerging threat to the health of vulnerable populations and the capacity to rapidly overload a health care service, resulting in potentially dramatic outcomes for patients [96, 97, 98]. TA describes an event of acute bronchospasm close in time to a local passage of a thunderstorm. It is now recognised that TA is a risk factor for asthma attacks in patients suffering from pollen allergy [99].

D'Amato et al. [99] predicted that climate change is likely to lead to more frequent and widespread episodes of thunderstorm asthma. One reason is that the frequency of thunderstorms is likely to increase with higher ocean and near‐surface warming [100]. Climate change also affects the growth patterns of allergen‐producing plants and the production (Section 2.2) and dispersal (Section 2.3) of allergenic pollen. This engages the risk of severe TA outbreaks.

Davies et al. [101] defined three components affecting the occurrence of TA: climate (thunderstorm, season), aeroallergens (grass pollen, fungal spores, and particulate matter), and individual characteristics (sensitisation to allergens, history of rhinitis/asthma, social factors, medication use). The occurrence of TA is associated with seasons with high atmospheric concentrations of airborne allergenic pollen and fungal spores [99]. In addition to Ryegrass pollen [102], some data implicate *Parietaria* pollen [99] or Olive tree pollen ( *Olea europaea* ) [103] to be related to TA.

The mechanisms of TA are not yet fully understood, but these events occur when certain meteorological and aerobiological factors combine [104]. During a thunderstorm, convective air masses transport pollen into the air, where they encounter humidity or precipitation. Because of osmosis and ionic charge differences caused by the thunderstorm, the pollen grains could break into fragments, which sink to the surface and can cause asthmatic reactions when inhaled [96, 97]. Further details on the bio‐physical mechanisms of TA will be found in D'Amato et al. [13] and Kevat [97].

So far, most TA cases have been reported from Australia [97]. In 2016, within 2 days more than 3400 TA‐related patients were treated in the emergency department in Melbourne. Consequently, this event had a very strong impact on the national public health system [96]. Moreover, studies showed that TA events also occur more frequently in other regions, for example, in different Bavarian cities in Germany [104, 105, 106] (Table 1).

**TABLE 1:** Research needs: Climate change affecting pollen allergies.

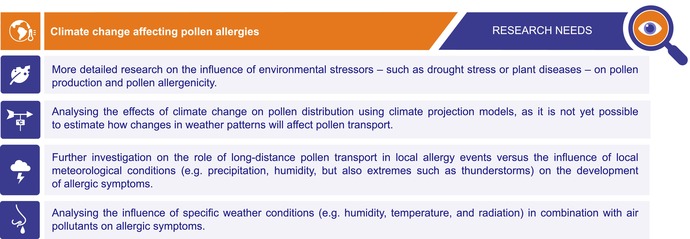

## 3 Climate Change Affecting Air Quality

The local air quality situation results from complex interactions of different processes. These are (1) local emissions, (2) atmospheric transport and influence of meteorological elements, (3) chemical processes, and (4) background concentrations of air pollutants and precursors, as well as (5) deposition. The variability of the air pollutant concentration, and thus of the air quality at a location, results from the dependency of the five processes. This dependency is different for the individual air pollutant.

### 3.1 Air Pollutants

#### 3.1.1 Dependence of Air Pollutant Concentrations on Meteorology

Emission sources are diverse and can be divided into two categories—natural (e.g., plants, oceans) and anthropogenic (e.g., traffic, industrial processes, agriculture, and households). Some of these sources depend directly or indirectly on the meteorological situation, for example, the emission of Biogenic Volatile Organic Compounds (BVOC) by trees and plants influenced by solar radiation and ambient air temperature [107]. The horizontal and vertical transport of air pollutants is highly dependent on wind (direction and velocity) and vertical mixing by turbulence. The latter is most intense in convective situations that are often related to high incoming solar radiation (heating of the ground). These transport processes influence the distribution of air pollutants on both small and large scales. Air pollutants underlie chemical transformations, resulting in production and reduction processes in the atmosphere, forming new gases as well as particles (gas‐to‐particle conversion). These processes typically depend on meteorological parameters such as temperature, radiation, and humidity, as well as on the concentration of other atmospheric pollutants. Gases, particles, and their transformation products are removed from the atmosphere by dry and wet deposition processes. The so‐called background concentration at a certain location is the result of emission, transport, distribution, and transformation of air pollutants in a large‐scale area, which is not affected by local emission sources.

#### 3.1.2 Sources and Sinks of Air Pollutants

Reactive trace gases [e.g., nitrogen oxides (NO_x_ = NO + NO_2_), and ozone (O_3_)] and particulate matter (PM) are considered the most relevant pollutants affecting air quality. Emissions of NO (the main part of emissions) and NO_2_ result from combustion processes for coal, oil, gas, wood, waste, and from road traffic, which is of great relevance in urban areas [108]. Ground‐level O_3_ is not emitted directly, but it is produced in the presence of solar radiation via photochemical processes from the precursor substances nitrogen oxides and volatile organic compounds (VOCs). Therefore, ozone is called a secondary pollutant. VOCs are emitted from a variety of anthropogenic (e.g., motor vehicles, chemical manufacturing facilities, and refineries) and natural sources (mainly trees, BVOC). High O_3_‐concentrations can reduce pulmonary function and result in lung diseases [109, 110, 111]. Plants suffer foliar damage, and long‐term exposure impairs growth and crop yield. Finally, PM consists of a heterogeneous mixture of solid and liquid particles suspended in the air that varies continuously in size and chemical composition in space as well as in time [112]. Compounds of particulate matter are, for example, organic matter (including pollen), elemental carbon, mineral dust, sea salt (primary particles), nitrate, ammonia, sulphate (secondary inorganic particles), and a mixture of different secondary organic particles. The fraction of PM with diameters ≤ 2.5 μm is named PM_2.5_ and is referred to as “fine particles”, the fraction from 2.5 to 10 μm is called PM_2.5–10_ and is typically referred to as “coarse particles”; and > 10 μm is referred to as “super coarse particles”. The coarse fraction consists mainly of primary particles, which are directly emitted from their sources (e.g., road traffic, power plants, heating of houses, metal, and steel production) into the atmosphere. Sources can also be natural, such as soil erosion (mineral dust), biomass burning, sea spray, and break‐up of larger solid biogenic particles (e.g., pollen, spores, plant, and insect parts). The PM_2.5_ fraction consists partly of primary sources but predominantly of secondary particles, which are produced in the atmosphere from gaseous precursors. An important source of the precursors of secondary particles is agriculture, especially ammonia emissions from livestock farming. Pollen grains, plant and insect parts, tyre and road abrasion, microplastics, and so forth cover a large size range. However, they are typically larger than 10 μm and therefore belong to the group of “super coarse particles”.

### 3.2 Direct Effects of Climate Change

A changing climate is anticipated to significantly affect meteorological conditions and thus has an impact on relevant processes for air quality, like an increase in average temperature, changes in the frequency and duration of heat waves, changes in precipitation, impact on cloud cover, and alterations in circulation patterns [20, 113, 114, 115]. These changes could result in a modification of natural and anthropogenic emissions and changes in chemical reaction rates, which are usually temperature‐dependent and often photochemical in the atmosphere. This also applies to the formation of secondary particles and heterogeneous reactions that take place at the phase boundary between gas and liquid phase (cloud droplets, aerosols). Changing meteorological parameters result in altered atmospheric residence times, and changes in mixing layer heights affect the dilution of pollutants.

The impact of climate change (via meteorological conditions, as shown in Figure 1) on concentrations of certain air pollutants, such as ozone, nitrogen dioxide, sulphur dioxide, and particulate matter, and their compounds has long been studied, similar to emission reduction effects [20]. Projected regional changes depend on a number of factors, such as emission reduction pathways for greenhouse gases and different anthropogenic emissions, or changes in land use and land cover for each climate region [116]. With mitigation measures in mind, the emission reductions must be included in all assessments of future air quality scenarios. However, although the behaviour of atmospheric air pollutants has been simulated under changed climatic conditions, there are still uncertainties, especially at combinations of climate change and changes in emissions, with focus on different regions as well as separated within these regions for urban and rural areas.

#### 3.2.1 Nitrogen Oxides

The atmospheric lifetime of nitrogen oxides strongly depends on radiation. Higher radiation leads to faster processing towards nitric acid; this would mean that less nitrogen trioxide (NO_3_) is available for the radical night‐time chemistry. Lightning could be enhanced in the future because of more convection in the troposphere [117]. Lightning is a large source of NO_x_ on a global scale and has an impact on tropospheric oxidation capacity (OH), especially in the tropics. Additionally, NO_x_ emissions indirectly affect Earth's radiative balance and thereby global climate [117, 118].

#### 3.2.2 Ground‐Level Ozone

As a result of mitigation strategies for ozone precursors, very high ozone concentrations are no longer as common as they were in the 1980s and 1990s. However, in urban areas, the very low ozone concentrations caused by so‐called titration effects also occur less frequently, so that in these regions, ozone concentrations have increased slowly [119] and the frequency distribution of ozone has become narrower overall. Long‐term model trends of tropospheric ozone show an increasing trend in the climate change prediction for the European summer climate [120, 121, 122]. This is mainly due to the predicted rise in temperature and the decrease in cloud cover, which leads to higher photochemical production of ozone from biogenic species. Droughts, which will also increase under future climate conditions, on the other hand, appear to contribute to a decrease in ozone concentrations as plants emit fewer ozone precursors. Fitzky et al. [123] summarised that the available studies show a consistent picture that tropospheric ozone concentrations are strongly influenced by climate change; however, the extent of the projected change of ozone is variable. The individual mechanisms and the complex relationship are far from being understood in detail, so there is a considerable need for research.

#### 3.2.3 Secondary Fine Particles

Secondary fine particles are formed from gaseous precursor substances (gas‐to‐particle conversion), for example, a significant proportion by ammonia, which is emitted by agriculture. It is expected that atmospheric ammonia (NH_3_) concentrations will increase because of climate change [124], leading to an increased formation of fine particles, if NO_x_ and sulphur dioxide (SO_2_) emissions remain at current levels. Secondary organic aerosols (SOA), which arise from the oxidation of anthropogenic or naturally emitted organic compounds, form another important fraction of secondary fine particles. The organic precursor substances are emitted more strongly because of increased temperatures, so that increased production is expected [125, 126].

#### 3.2.4 Primary Fine and Coarse Particles

The concentration of primary fine and coarse particles in the atmosphere is likely to rise due to increasing soil erosion [127] as a result of longer dry periods. In addition, the rise in temperature and drought will lead to an increase in forest and steppe fires [128], which will increase the amount of soot and opaque particles in the atmosphere. Although these sources increase, anthropogenic sources will decrease: Rising temperatures, especially in winter, will reduce the need for heating in households. Vehicles' combustion engines are typically equipped with exhaust after‐treatment systems, so that the internal engine sources of particulate matter are greatly reduced [129]. This does not apply to the coarse particles caused by (tyre‐, road‐, and brake‐) abrasion. Tyre wear is the largest source of microplastics [130]. Because of the steadily increasing total mileage of the fleet [131], and the trend towards heavier vehicles [132], these emissions are increasing. As part of the CO_2_ emission reduction measures, the vehicle fleet is becoming increasingly electrified. Electric vehicles have both a higher mass and higher acceleration than their counterparts equipped with combustion engines. Both lead to increased tyre and road abrasion. On the other hand, brake wear is lower because more vehicles brake wear‐free through recuperation. Global warming will also increase tyre abrasion. This is currently being investigated.

Although the health aspect is rather limited because of coarse mineral particles, an increased concentration of fine dust, and in particular an increased concentration of soot has a considerable impact on health. In addition to the health relevance of dust, there is also a pronounced inverse feedback on climate. Although mineral particles often exhibit a high degree of backscattering and thus counteract warming (e.g., sulphate aerosols), opaque particles and soot in particular have high light‐absorbing properties and thus intensify the greenhouse effect.

#### 3.2.5 Pollen

Pollen contributes to the particulate matter load in the atmosphere. As discussed in Section 2.1, higher temperatures might lead to extended periods with pollen load. Climate change enhances the emission and transport of coarse particles (among other pollen grains) to fine particles. The effect of the meteorological parameters on the individual components is different and still uncertain [133].

#### 3.2.6 Simultaneous Occurrence of Climate Change and Emission Changes

Not only changes in climate but also in emissions of pollutants and precursors are projected for the future. Changes in anthropogenic emissions occur over decades, and these might alter the average concentrations more than the effect of climate change [114]. The interaction between the changing meteorological parameters and the atmospheric chemical processes is not one‐way, but bidirectional. Changing atmospheric chemistry can have dampening as well as intensifying effects on climate change, e.g., the influence of particles and aerosols on the single scattering albedo and cloud formation [134]. Higher emissions of hydrocarbons and their subsequent photochemical degradation lead to increased water‐soluble oxidation products, for example, formaldehyde, which forms acetic acid very effectively via a complex, heterogeneous mechanism via the methan(edi)ol stage [135]. This process influences the acidification of precipitation.

The measures taken in spring 2020 to contain the COVID‐19 pandemic are a unique real‐life example of analysing the impact of emission reductions on pollutant concentrations and clearly show the complex influences that the current weather situation has on air pollutant concentrations. A first look at the measured values initially showed no influence of the lockdown on the pollutant concentrations at stations close to the traffic in Germany, since the reductions were almost completely masked by a simultaneously changing weather situation. Only when the meteorological effects were factored out was it found that the average reductions in NO_2_ concentrations measured at German urban monitoring stations near traffic were in the range of 20%–30% [136, 137], which can be attributed to the reduction in emissions. These results show that targeted air quality control measures in cities and the replacement of vehicle fleets are necessary, as they are the main cause of the significant decline in NO_2_ concentrations near traffic that has been observed for several years [136]. Deroubaix et al. [138] also confirmed a widespread NO_2_ concentration reduction for Europe during the March–May 2020 lockdown period. For O_3_, however, they found positive anomalies in northern and negative anomalies in southwestern Europe during the lockdown. This was mainly attributed to reduced cloudiness and enhanced radiation in regions with increased ozone formation and more clouds in regions with less ozone. Gaubert et al. [139] confirmed the combined effects of emission reductions and meteorological situation on secondary pollutants by global model studies for the COVID lockdown period. This shows that secondary reactions (e.g., ozone formation) are not directly related to emissions, but that meteorological effects might be of greater significance.

All these effects have not yet been adequately investigated, so that the net effects of climate change on air quality and the corresponding feedbacks can only be estimated with correspondingly large uncertainties.

### 3.3 Indirect Effects of Climate Change

Indirect effects are those where the cause‐and‐effect relationship is not direct or immediate. Indirect effects between climate change and air quality are, for example, changes in human behaviour. For example, warmer winters lead to less heating, which is associated with lower emissions. The increased use of renewable energies not only reduces nitrogen oxides and particulate matter but also lowers CO_2_ emissions, thus slowing down climate change. On the other hand, cooling is going to be an increasing issue in the summer time. In around 30 years, the global demand for cooling will be higher than the one for heating. As three times as much energy is currently required for cooling as for the same amount of heating this is a major challenge for the energy industry [140].

### 3.4 Effects of Future Emissions Scenarios

As described above, a consistent shift away from fossil fuels towards renewable energies leads to a reduction in emissions of air pollutants caused by combustion processes. On the other hand, replacing vehicles with combustion engines with electric drives leads to an increase in coarse particles (tyre abrasion/microplastics) [141]. It is also conceivable that H_2_ emissions will increase significantly because of leaks during transportation or refuelling as a result of the widespread use of hydrogen technology, for example [142]. Hydrogen influences methane and O_3_ formation in the troposphere and is oxidised to water in the stratosphere [143, 144]. Increasing emissions lead to an increase in water vapour content in the stratosphere, which in turn can lead to increased formation of PSCs (polar stratospheric clouds) and thus to ozone depletion [145]. Emissions of fine particles and soot have already risen regionally because of the increased use of so‐called comfort fireplaces with “renewable fuel” (wood) in residential areas. For the short term, this would be a negative development, as not only particulate matter and other air pollutants are emitted in greater extent but also more CO_2_ is emitted for the same amount of heat than with conventional oil or gas heating.

As the population, standard of living and industrialisation are expected to continue to increase, especially in the Global South, anthropogenic emissions might also continue to rise worldwide. The conversion of large areas of natural vegetation into farmland in combination with higher temperatures because of climate change, is leading to desertification in some parts of the world, meaning that higher particle emissions are to be expected (Table 2).

**TABLE 2:** Research needs: climate change affecting air quality.

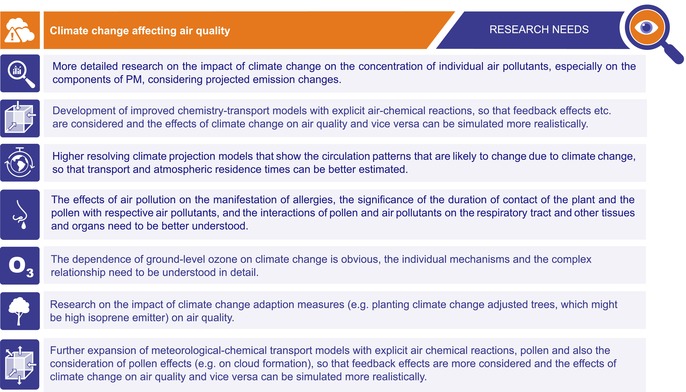

## 4 Air Pollutants Affecting Allergic Diseases

Reactive trace gases and particulate matter can impair allergenicity in different ways. Several studies shed light on the complex relationship between air pollution and the allergenicity of pollen grains [146, 147, 148]. The researchers observed changes in the proteome of allergen carriers, which could be attributed to the effects of air pollution. These changes could potentially contribute to the release of chemotactic substances, which could, in turn, increase the prevalence of allergies. Exposure to gaseous pollutants can also alter the quantity and timing of allergen release [24, 71, 149]. Through interaction with pollen grains and plant‐derived particles, pollutants can modify the morphology of allergen‐carrying agents, the pollen cell wall, the pollen protein content, or protein release from the pollen as well as the pollen protein itself [150, 151]. Not only do allergenic proteins play a role, but pollen‐derived lipids, called pollen‐associated lipid mediators (PALMs), which interact with the immune system, can modify the allergic reaction [152]. One particularly interesting observation was that substances such as PALMs were found to be more prevalent in pollen from urban areas [153, 154]. This finding suggests that urban areas may promote the development of allergies by increasing exposure to air pollutants, which not only act as proinflammatory triggers themselves but also enhance the allergenicity of pollen [154].

The mechanisms that could explain the enhanced sensitisation to aeroallergens by air pollutants include a greater antigenicity of proteins, increased deposition of allergen in the airways due to carriage by particles, increased epithelial permeability due to oxidative stress, and a possible direct adjuvant effect [155]. Additionally, the responses to air pollutants may vary among individuals because of, among others, genetic variations [154]. These findings have important implications for the development of allergy prevention and treatment strategies, as they highlight the need to consider a range of factors beyond simply identifying and avoiding allergens.

### 4.1 Influence of Trace Gases on the Allergenic Response and on the Allergenicity of Pollen

Controlled exposure studies on asthmatic patients have shown that NO_2_ can enhance the allergic response to inhaled allergens [151]. The respiratory mucosa formed by the airway epithelium represents the first contact between air pollutants and the respiratory system, functioning as a mechanical and immunologic barrier. Under conditions of air pollution exposure, the defence of the airway epithelium is compromised by the disruption of epithelial integrity, resulting in uptake of particles, activation of Toll‐like and NOD‐like receptors, epithelial growth factor receptors and induction of oxidative stress. Thus, inhalation of pollutants like nitrogen species (e.g., NO_2_) or ozone (O_3_) induces epithelial damage and inflammatory responses in the upper and lower airways, as shown by increased levels of inflammatory cells and mediators in nasal and bronchoalveolar lavage [156, 157, 158]. Furthermore, air pollutants could increase the risk of sensitisation and the responses to inhaled allergens in asthmatics [159]. Pollutants can act as adjuvants and affect the release of some cytokines (e.g., alarmines) of airway epithelial cells, which promote T‐helper 2 (Th2) phenotypic differentiation [156]. Such a potential enhancing effect has been demonstrated for NO_2_, O_3_, and SO_2_ [160].

Reactive pollutants, such as NO_2_ and O_3_ (Section 3.1), can facilitate the release of allergen‐rich cytoplasmic granules from pollen and therefore increase the quantity of allergens in the respirable submicronic fraction (PM < 1 μm) [161]. Moreover, both NO_2_ and O_3_ can lead to the nitration of airborne allergens, such as Bet v 1, the major allergen from birch ( *Betula pendula* ) [150, 162]. The detection of IgE specific for nitrated Bet v 1a, which does not bind unmodified Bet v 1 or nitrated unrelated proteins, implies that nitration generates novel allergenic epitopes. Interestingly, specific IgE for nitrated Bet v 1 is detected in serum samples of patients who are allergic to birch pollen, which indicates that allergen nitration is relevant in vivo and can contribute to allergenicity in polluted environments [23].

Nitration does not only induce nitration‐specific IgE but directly affects the allergenic potential of the birch pollen. Nitrated Bet v 1a results in stronger proliferation of Bet v 1‐specific T cell lines, and IgE binding to nitrated Bet v 1a is higher than IgE binding to Bet v 1 [163]. Because of nitration oligomerisation of Bet v 1 was also observed, which resulted in lower sensitivity to endosomal/lysosomal degradation [164].

Studies on ragweed ( *Ambrosia artemisiifolia* ) pollen showed higher allergen levels and increased IgE binding along high traffic roads compared to “vegetated areas”, with the higher IgE recognition being caused by recognition of the major allergen Amb a 1 [165]. Zhao et al. also showed an increased allergenicity of ragweed pollen and a direct link to increased human health risk and additional IgE binding to a new allergen in ragweed with homology to Hev b 9 from the rubber tree, induced by elevated NO_2_ concentrations [25, 166]. The fumigation of ragweed plants with elevated NO_2_ concentrations throughout a growing season resulted in increased overall S‐nitrosylation, and Amb a 1 is indicated as a possible candidate for S‐nitrosylation [25]. Other allergy‐relevant plants may be similarly affected, for instance, *Carpinus betulus* , *Ostrya carpinifolia*, and *Betula pendula* [150, 167].

High environmental ozone was associated with higher pathogen‐related proteins, as shown for Bet v 1. Pollen exposed to higher ozone levels was characterised by a higher immune stimulatory potential. Bet v 1 allergen content (PR‐10 protein) in the pollen was shown to be positively correlated with increasing ozone levels [153]. Furthermore, in *Cupressus arizonica*, an increase of the PR‐5 protein Cup a 3, a thaumatin‐like protein, was shown under polluted air conditions (polluted air areas in Barcelona and Madrid and unpolluted air areas in Gerona and Toledo were selected) [168]. Increased allergen contents due to elevated ozone have also been shown for other plant species, such as *Olea europaea* [169], *Lolium perenne*, and *Secale cereale* [170]. In addition, pollen wall modifications might affect immune reaction: in ozone‐fumigated ragweed pollen, reduced levels of wax compounds have been detected, and high ozone levels resulted in an altered lipid composition of birch pollen, which led to a modulated immune response [153].

High CO_2_ concentrations may facilitate faster growth of ragweed, earlier and more intense flowering, and a higher production of ragweed pollen [93]. Ragweed pollen allergenicity may be elevated through high atmospheric CO_2_ levels and increasing drought [94]. Besides, elevated ambient CO_2_ levels elicit a stronger RWE (aqueous ragweed pollen extract)‐induced allergic response in vivo and in vitro and RWE increased allergenicity depends on the interplay of multiple metabolites [171].

### 4.2 Influence of Particulate Matter on the Allergenic Potential of Pollen

Particulate matter can act directly on local antigen‐presenting cells, such as mucosal dendritic cells (DCs), and modulate their response by changing their surface phenotype and cytokine profile (reduced IL‐12 (Interleukin‐12) production), resulting in a proallergic, TH2 (T‐helper 2) dominated pattern of immune activation [160]. In addition, pollutants like diesel exhaust particles exacerbate allergic inflammation by increasing oxidative stress and neutrophilic infiltration—effects that can be mitigated by PGRN‐derived fragments, highlighting the immune‐amplifying role of air pollution [172].

Allergens from pollen are found in respirable particles (PM_10_), including whole anemophilous pollen grains (usually 10–100 μm in diameter) as well as pollen‐derived debris (0.6–2.5 μm in diameter) and submicronic particles called orbicules (0.02–1 μm in diameter) that possess high allergenic potential and can act as potent triggers of allergic airway inflammation, including bronchial asthma [173]. As described above, particles of 100–10 μm in diameter deposit in the upper respiratory tract, particles of 10–2.5 μm settle in the trachea, primary and secondary bronchia, whereas fine particles < 2.5 μm reach the alveoli [174, 175]. Pollen is affected by various factors, such as air particles, and its behaviour can change depending on weather conditions. These factors also influence the movement and spread of pollen and fungal spores in the air. Although many more studies are needed on the influence of PM on the production and allergenic potential of pollen, the reduction of PM, especially the fine fraction, is imperative to prevent and mitigate allergic respiratory inflammations. Emerging evidence suggests that in urban environments with elevated levels of air pollution, there is a marked increase in respirable pollen‐derived particles, highlighting a previously underestimated interface between aerobiological exposure and environmental toxicology.

The climate‐driven particle emissions (Section 3.1) may alter pollen and spore surfaces' physicochemical characteristics with effects on their allergenic potential [112]. In polluted areas, the interaction between pollen and particles results in quantitative and qualitative alteration of aeroallergens. Seasonal high PM loading in the urban and industrial atmosphere coincides with aeroallergen‐promoting micro‐ to nanoparticles' attachment to pollen's surface. A high frequency of positive sensitisation to pollen with particle loading was detected, suggesting that particle emissions may alter pollen surfaces' physicochemical characteristics with further consequences for their allergenic potential [112]. Airborne particles can mediate agglomeration of particles onto pollen surfaces, followed by pre‐activation of pollen, which then may induce local allergen release under appropriate conditions (humidity, etc.) [176].

Diesel exhaust Particles (DEP), specifically PM_10_, PM_2.5_, and ultrafine particulate matter (UFP), have been extensively investigated for their capacity to enhance Th2‐directed immune responses in humans. Intranasal exposure to DEP during allergen exposure (e.g., ragweed) was shown to increase local Th2 cytokine and specific IgE production. Inhalation of DEP at environmentally relevant concentrations augments allergen‐induced allergic inflammation in the lower airways of atopic individuals [177]. Moreover, DEP exposure increased the risk of early aeroallergen sensitisation, associated with allergic rhinitis by the age of 4 [178]. DEP‐induced oxidative stress plays a central role in this process [156]. As allergic sensitisation and the elicitation of symptoms are dose‐dependent phenomena, factors that modulate the bioavailability of allergens can influence allergenicity, too [179]. Maybe in the future, DEP will play a less prominent role because of exhaust gas treatment and increasing electrification of the vehicle fleet.

### 4.3 Effect of Climate Change on Pollen‐Food Allergy Syndrome

Importantly, sensitisation to pollen, particularly tree pollen, is considered a major risk factor for the development of pollen‐food allergy syndrome (PFS). Initially thought to predominantly affect adults, emerging evidence suggests that PFS is now becoming more prevalent in children, potentially as a consequence of the rising rates of SAR. Although PFS is generally regarded as a mild condition, it can lead to severe allergic reactions in some cases. Furthermore, a wide range of plant‐based foods can trigger symptoms, which could negatively affect dietary choices and nutritional intake, particularly for those affected. In conclusion, the increasing rates of SAR, driven by climate change, are likely contributing to a rise in PFS cases. This underscores the need for greater awareness and research into how climate change is influencing allergic diseases and food‐related allergies [180] (Table 3).

**TABLE 3:** Research needs: climate change affecting allergic diseases.

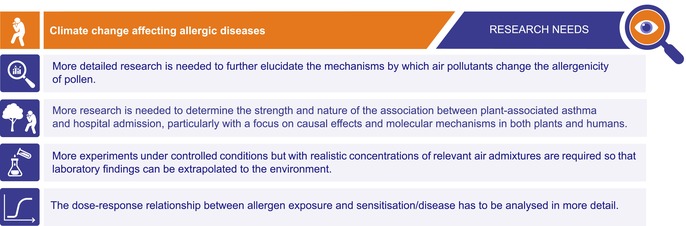

## 5 Comparison of Urban and Rural Areas

Besides climate and air quality, biodiversity and land use are closely associated with pollen allergies. However, these associations are not always the same in urban and rural areas. A region‐specific perspective is therefore needed.

### 5.1 Specifics in Urban Areas

Climate change leads to numerous additional problems and challenges specific to urban conditions. Increased night‐time temperatures (urban heat islands) [28] and air pollution are two of these [29]. Furthermore, the effects of changes in the urban environment are difficult to predict in terms of their impact on human health, making things even more complicated [93, 181].

Changes of plants and pollutant exposure take place in different areas, including the personal vicinity. This is due to intentional changes as well as unintentional consequences of actions. The authors of numerous studies advocate systematic greening of urban areas and emphasise the extensive services provided by urban green spaces to preserve biodiversity and human health as for example heat mitigation, improving air quality through filtration of polluted air [182], protecting against flash flooding during extreme precipitation events [183, 184, 185, 186, 187].

Urban vegetation is therefore usually seen as positive for health and wellbeing, but in certain cases, it can also have adverse or at least undesired effects. Although urban greening is widely promoted as a nature‐based solution for improving public health, climate resilience, and air quality, it can also present a paradox in the context of allergen exposure. Depending on the plant species selected and how these green spaces are managed, they can contribute to increased airborne pollen concentrations of certain pollen types. If the pollen is (potentially) allergy‐relevant, this can raise the allergy burden in the population. This is particularly relevant in densely populated areas, where large numbers of individuals may either already suffer from allergic rhinitis or asthma, or may become newly sensitised. The key challenge is designing green infrastructures that balance ecological, climatic, and health‐related objectives without inadvertently increasing exposure to allergenic pollen. Therefore, the allergenicity of plants should be considered alongside their tolerance to environmental stressors, biodiversity value, and maintenance requirements as important criteria in the selection and management of plant species in urban environments. As an example, large trees along busy city streets are useful in reducing UV exposure, but the reduced mixing of exhaust‐contaminated air can lead to an increase in the local air pollution levels [28]. Moreover, pollen allergens can be distributed very diversely within the urban environment, and local small‐scale conditions can have a significant influence on pollen exposure at the ground level [182]. Even though large trees are helpful and desired in cities, vertical ventilation of the ground area should be taken into consideration.

In the selection of plants, the needs of allergy sufferers are hardly considered so far. Instead, attention is usually paid to the robustness of plants against environmental stressors (e.g., climatic tolerance against heat or drought), to ecological or aesthetical aspects, to costs for maintenance, or to cultural values. Current trends are often influenced by landscape architects or home and garden magazines, and shops. Only recently, their role as anti‐pollution measures as well as their allergenic potential is playing a role in planning, remodelling, and redesigning green spaces [188, 189].

The following measures should be considered when planning green spaces in urban areas:

- Planning should include the special characteristics of flora, resulting from current and future climatic conditions, as well as the conditions of the soil.
- Dominance and overabundance of plant species with (potentially) allergenic pollen should be avoided.
- Preventing grasses from flowering and pollen release by lawn mowing should be done (inner cities, front yards, residential areas with detached houses, urban parks) before flowering in order to reduce the grass pollen load.
- The promotion of areas with high biodiversity as a concept of “urban wilderness” should be done with careful consideration of the allergenicity of the vegetation. In the vicinity of kindergartens, schools, or hospitals wilderness‐related concepts with a potential dominance of plants with allergenic pollen are not suitable.

The spread of alien plant species (with allergenic pollen) like ragweed ( *Ambrosia artemisiifolia* ) should be prevented on the one hand by appropriate control measures by the municipalities and on the other hand by educating the public in order to increase awareness of the problem.

The management of urban green spaces, whether targeted or not, whether municipal or private, influences the frequency and intensity of contact with allergenic pollen, as well as the concentration of pollutants and the urban climate. In this context, the influence and effectiveness of management should be investigated with regard to the expected effects on allergy sufferers, for example, by calculating allergenicity indexes and carrying out an allergic risk assessment [190, 191, 192, 193].

Numerous recent studies on allergy risk in urban landscapes conclude that pollen allergenicity and pollination characteristics of vegetation should be included as key parameters in the design, planning, and management of current and future green spaces [188, 193, 194, 195].

At the same time, the resistance of urban vegetation to future climate change must be considered to preserve the function of urban greenery in the long term. Research into the cultivation of species adapted to climate change is an urgent task for the coming years. In this context, it might be necessary to review and discuss the requirements of some alien species, as they can make a valuable contribution to the conservation of green spaces under increasingly extreme environmental conditions in the future [196, 197].

However, it should be kept in mind that the spectrum of urban‐adapted tree species is relatively small and that, whether intentionally or unintentionally, they are becoming established in urban environments worldwide and homogenising the urban flora [198, 199, 200]. This can mean that allergy sufferers when travelling to other countries may encounter the same pollen allergens as they do at home, making spatial avoidance of allergens during pollen season difficult.

Another aspect is the preservation of biodiversity. Reducing the mowing frequency has been shown to increase the ecological value of lawns for flora and fauna [201, 202], but on extensive meadows, grasses come to flower intensively and more often, and thereby increase the number of grass pollen released.

### 5.2 Specifics in Rural Areas

Rural areas are typically a more or less intensively managed cultural landscape. Nature reserves are very rare in densely populated countries. Despite their distance from urban areas, rural landscapes are an enormous source of pollen production affecting residents of rural as well as urban areas. The latter is attributed to transport by wind (Section 2.3). Rural areas are also affected by, for example, extreme weather events and droughts, which will occur more frequently as a result of climate change, especially in summer. Ozone concentrations are usually higher in rural areas than in cities coinciding with higher pollen concentrations in rural areas [203]. This could be associated with a higher health risk for the rural population, but further research is needed.

In general, rural areas are characterised by less fragmentation and a relative consistency of certain land uses as pastures, grassland, or forest. Although rural areas do not usually represent the “natural” vegetation, they represent a higher “nature feeling” for most people and therefore have a recreational value. They often represent their own unique and distinctive features and patterns that differ significantly from those in urban areas. In addition, the number of exotic plant species is significantly lower than in urban areas. Rural or near‐natural areas can be affected by the growth of cities and towns. Roads and other infrastructure measures can lead to the fragmentation of habitats and the sealing of soil [204]. Fertilisation, whether intentional or resulting from environmental nitrogen deposition, can alter plant communities by favouring competitive, nutrient‐demanding species. This may reduce plant species diversity and influence both the composition of the airborne pollen spectrum and the magnitude of concentration of certain pollen types. In a recent study by Daelemans et al. [205], nitrogen‐enriched grasslands were found to have higher grass (Poaceae) cover and greater overall pollen quantity compared to non‐enriched common semi‐natural grasslands. Additionally, pollen from fertilised grasslands showed increased allergenic potential. Although the role of fertilisation of plants in allergen exposure was not a primary focus of our position paper, its potential relevance warrants further investigation, as also recommended in Daelemans et al. [205].

Climate change will lead to distinct changes in rural areas: especially extreme weather events like heavy rainfall and flooding, as well as droughts, cause erosion and desertification. More intense and prolonged heat and drought periods can lead to the decline of previous vegetation and the growth of new vegetation that may be dominated by plants with allergenic pollen. Agricultural areas that are in preparation for planting and not yet covered with vegetation may lead to more frequent dust formation in the future and thus to an increase in PM concentrations. Air pollution, especially PM_10_, could be exacerbated by increasing wildfires, which could lead to increased health risks for exposed populations [206, 207]. Wildfires are increasingly occurring because of climate change, both in number and in affected area. This situation will worsen further as climate change progresses. For example, the Canadian wildfire season in 2023 set a decades‐long record. In addition to higher CO_2_ emissions, air quality deteriorated, particularly regarding components such as fine dust and soot. Emissions from the Canadian wildfires worsened air quality in Eastern Canada, the Northeastern United States, were transported across the Atlantic, and could also be detected in Western Europe [208].

Because rural areas offer a much larger area available for plant growth compared to urban areas, changes in land use of rural areas can have a large impact on the amount of pollen released, which can affect urban residents as well [209]. Examples are:

- Woodland: The conversion of monoculture forests to mixed forests can reduce the overabundance of some pollen types in the air [210, 211]. However, the effects of pollen from tree species with allergic potential, such as *Betula*, *Ostrya carpinifolia*, or some members of the *Cupressaceae* family, on the general population should be considered when converting forests. This should also be considered in large‐scale reforestation of previously deforested areas (e.g., because of bark beetle outbreaks or drought damage).
- Pastures and meadows as well as cropland that are being (purposely) set‐aside [212] can have a potential impact on the occurrence of certain pollen types in the airborne pollen spectrum. These areas may have higher occurrences of pollen from ruderal plants such as *Artemisia*, *Chenopodiaceae*, or *Plantago*. Re‐cultivation of previously set‐aside areas would have an opposite effect: The number of pollen from ruderal species would decrease, and the number of pollen from cultivated plants, for instance, from the genus *Brassica* (e.g., *Brassica napus* ) or *Secale* would increase.
- If cropland is expanded at the expense of permanent grassland, less grass or herb pollen can be expected. The same trend can be seen when grassland is used very intensively by livestock (fewer grasses reach the flowering stage) [213]. The abandonment of grassland farming in difficult‐to‐farm areas (e.g., alpine pastures) can also have a potential impact on pollen occurrence: In these cases, less grass pollen is expected and is replaced by more tree pollen [214].

New or exotic pollen emitters can either migrate slowly (e.g., *Ambrosia artemisiifolia* ) or spread more rapidly if planted (e.g., *Cupressus sempervirens* ) or cultivation/planting is encouraged (e.g., crops for energy production, rapeseed ( *Brassica napus* ), and maize ( *Zea mays* )) [215]. Planting of other pollen emitters can also be prohibited (such as in the case of *Ailanthus altissima* ), and their spread limited by legal restrictions on invasive plant species [212].

In addition, globalisation and climate change are leading to the spread of plant diseases and pests [209, 216], that have the potential to reduce the size of existing pollen sources locally or regionally [215, 217, 218, 219, 220, 221, 222] (Table 4).

**TABLE 4:** Research needs: comparison of urban and rural areas.

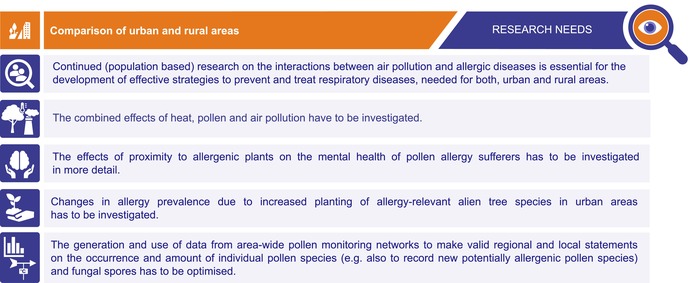

## 6 Conflict of Goals

Measures to mitigate climate change and improve air quality have conflicting objectives with regard to the health and quality of life of the population. Especially in cities, green spaces play an important role and have a positive impact on the health and quality of life. In addition, urban green spaces are important for the bioclimate of urban areas, which become even more important in the context of climate change. Against this background, the expansion of green spaces is a concern for many municipalities.

Depending on their composition, urban green spaces can cause or exacerbate negative health effects, for example, by emitting aeroallergens that lead to health problems. Exposure to allergenic pollen must therefore be considered in the identification and design of public green spaces. When selecting plants for greening, the top priority must therefore be low allergy and the second priority must be their resilience to the factors of climate change.

In addition, biogenic VOC emissions can contribute to the formation of ozone, which also has negative health effects (Section 3). The most important BVOC emissions for ozone formation are isoprene emissions from some tree species. German cities are currently changing the mix of tree species to make them more resilient to climate change. However, many of the tree species that have recently been planted for climate resilience reasons are strong emitters of isoprene (e.g., plane tree) and thus contribute to downwind ozone formation. With an expected decrease in nitrogen oxide emissions by mid‐century (e.g., through the electrification of the vehicle fleet), ozone production will develop in the direction of NO_x_ limitation, so that higher BVOC emissions will no longer have any or at least no proportional effect on ozone production.

Another conflicting goal is the increased use of biomass as a renewable and, therefore, supposedly climate‐neutral energy source. In addition to the significantly higher CO_2_ emissions compared to fossil fuels comfort fireplaces emit significant amounts of particulate matter and soot as well as polycyclic aromatic hydrocarbons (PAHs), which are harmful to health. In populated areas, a challenging concerted switch to emission‐free heating and cooling systems is required. For example, the use of air‐to‐water heat pumps in less densely populated areas and of waste water heat pumps connected to the sewage network in densely populated areas are conceivable here. This could generate heat in winter and cold in summer with high efficiency.

Replacing internal combustion vehicles with electric vehicles will gradually reduce local emissions of nitrogen oxides and PM_10_, but will increase emissions of coarse particulates because of increased tyre and road wear. In cities, the consistent switch to climate‐friendly modes of transport, namely cycling, walking, and electrified public transport, can solve the problem. Two good examples are today already Copenhagen and Paris.

## 7 Main Recommendations of the Expert Panel

Pollen allergies are one of the major health issues worldwide. Climate change is affecting many aspects of our environment, thereby influencing the occurrence, frequency, and severity of allergies. There are numerous studies focusing on the influence of climate change and pollution on pollen and allergies; nevertheless, there are many unanswered questions—particularly regarding the combined effects of multiple environmental stressors. Further research and coordinated action are essential to improve allergy management and deepen our understanding of these complex interactions. Although guideline‐based asthma management can improve individual patient outcomes, only regulatory measures ensuring cleaner air will lead to a sustained reduction in the overall burden of allergic disease [223] (Table 5).

**TABLE 5:** Recommendations for action.

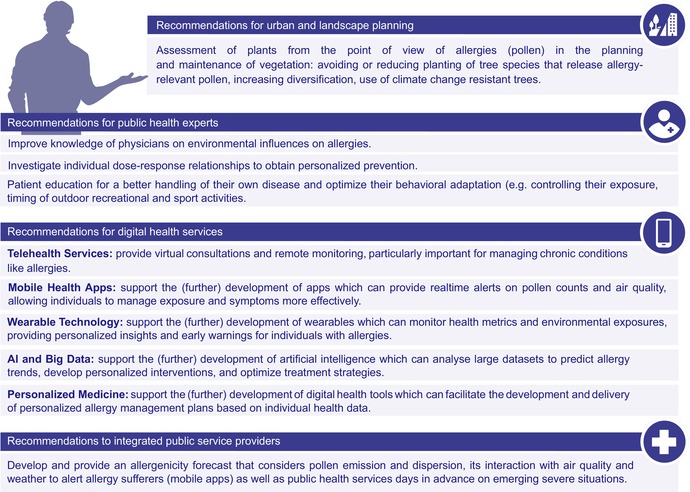

## Author Contributions

All authors had substantial contributions to the analysis or interpretation of data for this paper, revised it critically for important intellectual content, approved it finally, and agreed to be accountable for this work in ensuring its integrity of interpretation of data.

## Funding

The authors acknowledge financial support from the Open Access Publication Fund of UKE—University Medical Center Hamburg‐Eppendorf.

## Conflicts of Interest

The authors J.A., S.G., H.A., U.D., C.E., R.H., C.H., W.K., K.H.S., W.S., B.W., M.W., and C.T.‐H. have no conflicts of interest to declare. T.Z. has served as a consultant, researcher, and/or has received research grants from companies including: Bayer Health Care, FAES, Novartis, Henkel, AstraZeneca, AbbVie, ALK, Almirall, Astellas, Bayer Health Care, Beiersdorf, Bencard, Berlin Chemie, HAL, Leti, Meda, Menarini, Merck, MSD, Novartis, Pfizer, Sanofi, Stallergenes, Takeda, Teva, UCB, Henkel, Kryolan, and L'Oréal.

## Supporting information


**Appendix S1:** all70159‐sup‐0001‐AppendixS1.docx.

## Data Availability

The data that support the findings of this study are available from the corresponding author upon reasonable request.
