# Supplementary material for: Climate Change, Air Quality, and Pollen Allergies—State of the Art and Recommendations for Research and Public Health
Source: Allergy. 2025 Dec 20;81(3):663–83. doi: 10.1111/all.70159 (PMC12954572; doi:10.1111/all.70159)
Supplement: Supplementary file 1 — Appendix S1: all70159‐sup‐0001‐AppendixS1.docx. [file ALL-81-663-s001.docx]

**Supplement**

**Table S1.** Onset of flowering (phenological observation) of selected plant taxa or the beginning of pollen season (defined by pollen concentration in the air) of selected pollen types in different regions.

| **Plant taxon or pollen type** | **Change in the onset of flowering or the beginning of pollen season (reference period)** | **Area** | **Reference** |
| --- | --- | --- | --- |
| Hazel | change in the onset of flowering: 30 days earlier (1951–2021) | Germany | DWD |
|  | beginning of pollen season: 21 days earlier (1973–2013) | Sweden | Lind et al., 2016^1^ |
|  | 25 days earlier (1969–2018) | Switzerland | Frei, 2020^2^ |
|  | 9-18 days earlier (1990–2020) | Switzerland | Glick et al., 2021^3^ |
|  | 14 days earlier (1982–2015) | Belgium | Hoebeke et al., 2018^4^ |
|  | earlier start (2000–2016) | Italy | Cristofolini et al., 2020^5^ |
| Alder | 26 days earlier (1951–2021) | Germany | Phenological observations (annual reporters), DWD |
|  | 4-13 days earlier (1990–2020) | Switzerland | Glick et al., 2021^3^ |
|  | 10 days earlier (1973–2013) | Sweden | Lind et al., 2016^1^ |
|  | not significantly earlier start (2002–2019) | Slovakia (Bratislava) | Ščevková et al., 2021^6^ |
|  | not significantly later start (2004–2018) | Spain | Rojo et al., 2021^7^ |
|  | 3 days earlier (1982–2015) | Belgium | Hoebeke et al., 2018^4^ |
|  | no significant changes (1996–2014) | Italy (Turin) | Novara et al., 2016^8^ |
|  | not significantly later start (1995–2011) | Italy (Perugia), Iberian Peninsula (Vigo, Ourense) | Jato et al., 2013^9^ |
| Birch | 9 days earlier (1991–2021) | Germany | Phenological observations (annual reporters), DWD |
|  | 6 days earlier, not significant (1988–2018, except 1995) | Germany (Munich) | Bergmann et al. 2020^10^ |
|  | 15 days earlier (1973–2013) | Sweden | Lind et al., 2016^1^ |
|  | 10 days earlier (1882–2015) | Belgium | Hoebeke et al., 2018^4^ |
|  | 10 days earlier (1969–2018) | Switzerland (Basel) | Frei, 2020^2^ |
|  | no changes (1990-2020) | Switzerland | Glick et al., 2021^3^ |
|  | no clear trend (2000–2016) | Italy | Cristofolini et al., 2020^5^ |
|  | not significantly later start (1995–2011) | Italy (Perugia), Iberian Peninsula (Vigo, Ourense) | Jato et al., 2013^9^ |
|  | no significant trend (1995–2020) | United Kingdom | Adams-Groom et al., 2022^11^ |
| Grasses | 13 days earlier (1991–2021), meadow fox tail | Germany | Phenological observations (annual reporters), DWD |
|  | 14 days earlier (1982–2015) | Belgium | Hoebeke et al., 2018^4^ |
|  | less than 3 days earlier (1973–2013) | Sweden | Lind et al., 2016^1^ |
|  | 14 days earlier (1982–2015) | Belgium | Hoebeke et al., 2018^4^ |
|  | 6 days earlier (1969–2018) | Switzerland (Basel) | Frei, 2020^2^ |
|  | 8-25 days earlier (1990–2020) | Switzerland | Glick et al., 2021^3^ |
|  | earlier start (2000–2016) | Italy | Cristofolini et al., 2020^5^ |
|  | 4 days earlier (1978–2018) | Spain (Madrid) | Subiza et al., 2022^12^ |
|  | not significantly later start (2002–2019) | Slovakia (Bratislava) | Ščevková et al., 2021^6^ |
|  | no significant trend (1995–2020) | United Kingdom | Adams-Groom et al., 2022^11^ |
| Mugwort | no trend (1991–2021) | Germany | Phenological observations (annual reporters), DWD |
|  | no clear trend (1990–2020) | Switzerland | Glick et al., 2021^3^ |
|  | 12 days earlier (1973–2013) | Sweden | Lind et al., 2016^1^ |
|  | 7 days earlier (1982–2015) | Belgium | Hoebeke et al., 2018^4^ |
|  | not significantly later start (2002–2019) | Slovakia (Bratislava) | Ščevková et al., 2021^6^ |
|  | later start (2000–2016) | Italy | Cristofolini et al., 2020^5^ |

Definition of pollen season used in the publications listed in the Table S1.

Definitions for the beginning and end of pollen season based on percentages of the accumulated sum of daily pollen counts/m³ from the annual total:

a) 2.5% and 97.5%.^2,5,9,11,13^

b) 3% and 97%.^1^

c) 5% and 95%.^4,8^

Definitions for the beginning and end of pollen season based on certain thresholds:

a) first (last) day with a daily average ≥ 10 pollen/m^3^.^6^

b) first (last) of three consecutive days with >10 pollen/m^3^.^12^

c) EAACI definition by Pfaar et al.^10,14^

Six different pollen season definitions – two percentage-based and four threshold-based.^3^

**Table S2**. Changes in the Annual Pollen Integral (APIn) or Seasonal Pollen Integral (SPIn) for selected pollen taxa in different regions. Definition of Annual Pollen Integral (APIn) and Seasonal Pollen Integral (SPIn) was based on Galan.^19^

| **Plant**  **species** | **Length of time series (data period)** | **Change in Annual or Seasonal Pollen Integral** | **Area** | **Reference** |
| --- | --- | --- | --- | --- |
| Hazel | 40 years (1981–2020) | increase | Benelux | de Weger et al., 2021^15^ |
|  | 41 years (1973–2013) | significant increase | Sweden | Lind et al., 2016^1^ |
|  | 31 years (1990–2020) | significant increase | Switzerland | Glick et al., 2021^3^ |
|  | 50 years (1969–2018) | significant increase | Basel (Switzerland) | Gehrig & Clot, 2021^16^ |
| Alder | 40 years (1981–2020) | significant increase | Benelux | de Weger et al., 2021^15^ |
|  | 41 years (1973–2013) | increase | Sweden | Lind et al., 2016^1^ |
|  | 24 years (1994–2017) | increase | Spain (South) | Velasco-Jiménez et al., 2020^17^ |
|  | 50 years (1969–2018) | significant increase | Basel (Switzerland) | Gehrig & Clot, 2021^16^ |
|  | 10–30 years (max. 1989–2018) | clear increase | Bavaria (German) | Rojo et al., 2021^13^ |
| Birch | 40 years (1981–2020) | significant increase | Benelux | de Weger et al., 2021^15^ |
|  | 41 years (1973–2013) | increase | Sweden | Lind et al., 2016^1^ |
|  | 31 years (1990–2020) | significant increase | Switzerland | Glick et al., 2021^3^ |
|  | 50 years (1969–2018) | increase | Basel (Switzerland) | Gehrig & Clot, 2021^16^ |
|  | 10-30 years (max. 1989–2018) | clear increase | Bavaria (German) | Rojo et al., 2021^13^ |
|  | 26 years (1995–2020) | clear increase | United Kingdom | Adams-Groom et al., 2022^11^ |
| Grasses | 40 years (1981–2020) | significant decrease | Benelux | de Weger et al., 2021^15^ |
|  | 41 years (1973–2013) | increase | Sweden | Lind et al., 2016^1^ |
|  | 50 years (1969–2018) | no changes | Basel (Switzerland) | Gehrig & Clot, 2021^16^ |
|  | 10-30 years (max. 1989–2018) | significant decrease | Bavaria (German) | Rojo et al., 2021^13^ |
|  | 40 years (1978–2018) | slight decrease | Spain (Madrid) | Subiza et al., 2022^12^ |
|  | 26 years (1995–2020) | no significant trend | United Kingdom | Adams-Groom et al., 2022^11^ |
|  | 21 years (1998–2018) | SPI: significant increase | Iceland (Akureyri) | Przedpelska-Wasowicz et al., 2021^18^ |
|  | 31 years (1988–2018) | SPI: not significant decrease | Iceland (Reykjavík) | Przedpelska-Wasowicz et al., 2021^18^ |
| Mugwort | 40 years (1981–2020) | significant decrease | Benelux | de Weger et al., 2021^15^ |
|  | 41 years (1973–2013) | increase | Sweden | Lind et al., 2016^1^ |
|  | 50 years (1969–2018) | significant decrease | Basel (Switzerland) | Gehrig & Clot, 2021^16^ |

.

**References**

1. Lind T, Ekebom A, Alm Kübler K, Östensson P, Bellander T, Lõhmus M. Pollen Season Trends (1973-2013) in Stockholm Area, Sweden. *PLoS One*. 2016;11(11):e0166887. doi:10.1371/journal.pone.0166887

2. Frei T. Climate change in Switzerland: Impact on hazel, birch, and grass pollen on the basis of half a century of pollen records (1969 - 2018). *Allergol Select*. 2020;4:69-75. doi:10.5414/ALX02180E

3. Glick S, Gehrig R, Eeftens M. Multi-decade changes in pollen season onset, duration, and intensity: A concern for public health? *Sci Total Environ*. 2021;781:146382. doi:10.1016/j.scitotenv.2021.146382

4. Hoebeke L, Bruffaerts N, Verstraeten C, et al. Thirty-four years of pollen monitoring: an evaluation of the temporal variation of pollen seasons in Belgium. *Aerobiologia*. 2018;34(2):139-155. doi:10.1007/s10453-017-9503-5

5. Cristofolini F, Anelli P, Billi BM, et al. Temporal trends in airborne pollen seasonality: evidence from the Italian POLLnet network data. *Aerobiologia*. 2020;36(1):63-70. doi:10.1007/s10453-019-09609-8

6. Ščevková J, Dušička J, Hrabovský M, Vašková Z. Trends in pollen season characteristics of Alnus, Poaceae and Artemisia allergenic taxa in Bratislava, central Europe. *Aerobiologia*. 2021;37(4):707-717. doi:10.1007/s10453-021-09717-4

7. Rojo J, Fernández-González F, Lara B, et al. The effects of climate change on the flowering phenology of alder trees in southwestern Europe. *Mediterr Bot*. 2021;42:e67360. doi:10.5209/mbot.67360

8. Novara C, Falzoi S, La Morgia V, Spanna F, Siniscalco C. Modelling the pollen season start in Corylus avellana and Alnus glutinosa. *Aerobiologia*. 2016;32(3):555-569. doi:10.1007/s10453-016-9432-8

9. Jato MV, Rodríguez-Rajo FJ, Aira MJ, Tedeschini E, Frenguelli G. Differences in atmospheric trees pollen seasons in winter, spring and summer in two European geographic areas, Spain and Italy. *Aerobiologia*. 2013;29(2):263-278. doi:10.1007/s10453-012-9278-7

10. Bergmann K-C, Buters J, Karatzas K, et al. The development of birch pollen seasons over 30 years in Munich, Germany-An EAACI Task Force report. *Allergy*. 2020;75(12):3024-3026. doi:10.1111/all.14470

11. Adams-Groom B, Selby K, Derrett S, et al. Pollen season trends as markers of climate change impact: Betula, Quercus and Poaceae. *Sci Total Environ*. 2022;831:154882. doi:10.1016/j.scitotenv.2022.154882

12. Subiza J, Cabrera M, Jm C-R, Jc C, Mj N. Influence of climate change on airborne pollen concentrations in Madrid, 1979-2018. *Clin Exp Allergy*. 2022;52(4):574-577. doi:10.1111/cea.14082

13. Rojo J, Picornell A, Oteros J, et al. Consequences of climate change on airborne pollen in Bavaria, Central Europe. *Reg Environ Change*. 2021;21(1). doi:10.1007/s10113-020-01729-z

14. Pfaar O, Bastl K, Berger U, et al. Defining pollen exposure times for clinical trials of allergen immunotherapy for pollen-induced rhinoconjunctivitis - an EAACI position paper. *Allergy*. 2017;72(5):713-722. doi:10.1111/all.13092

15. Weger LA de, Bruffaerts N, Koenders MMJF, et al. Long-Term Pollen Monitoring in the Benelux: Evaluation of Allergenic Pollen Levels and Temporal Variations of Pollen Seasons. *Front Allergy*. 2021;2:676176. doi:10.3389/falgy.2021.676176

16. Gehrig R, Clot B. 50 Years of Pollen Monitoring in Basel (Switzerland) Demonstrate the Influence of Climate Change on Airborne Pollen. *Front Allergy*. 2021;2:677159. doi:10.3389/falgy.2021.677159

17. Velasco-Jiménez MJ, Alcázar P, La Díaz de Guardia C, et al. Pollen season trends in winter flowering trees in South Spain. *Aerobiologia*. 2020;36(2):213-224. doi:10.1007/s10453-019-09622-x

18. Przedpelska-Wasowicz EM, Wasowicz P, Áskelsdóttir AÓ, Guðjohnsen ER, Hallsdóttir M. Characterisation of pollen seasons in Iceland based on long-term observations: 1988–2018. *Aerobiologia*. 2021;37(3):507-524. doi:10.1007/s10453-021-09701-y

19. Galán C, Ariatti A, Bonini M, et al. Recommended terminology for aerobiological studies. *Aerobiologia*. 2017;33(3):293-295. doi:10.1007/s10453-017-9496-0
